# Synthesis and Structural Analysis of High‐Silica ERI Zeolite with Spatially‐Biased Al Distribution as a Promising NH_3_‐SCR Catalyst

**DOI:** 10.1002/advs.202307674

**Published:** 2024-02-02

**Authors:** Jie Zhu, Koki Muraoka, Takeshi Ohnishi, Yutaka Yanaba, Masaru Ogura, Akira Nakayama, Toru Wakihara, Zhendong Liu, Tatsuya Okubo

**Affiliations:** ^1^ Department of Chemical System Engineering The University of Tokyo 7‐3‐1 Hongo Bunkyo‐ku Tokyo 113‐8656 Japan; ^2^ Institute of Industrial Science The University of Tokyo 4‐6‐1 Komaba Meguro‐ku Tokyo 153‐8505 Japan; ^3^ Institute of Engineering Innovation The University of Tokyo 2‐11‐16 Yayoi Bunkyo‐ku Tokyo 113‐8656 Japan; ^4^ State Key Laboratory of Chemical Engineering Department of Chemical Engineering Tsinghua University Haidian District Beijing 100084 China

**Keywords:** density functional calculations, high‐silica ERI zeolite, NH3‐SCR catalyst, NMR spectroscopy, spatially‐biased Al distribution

## Abstract

Erionite (ERI) zeolite has recently attracted considerable attention for its application prospect in the selective catalytic reduction of NO_x_ with NH_3_ (NH_3_‐SCR), provided that the high‐silica (Si/Al > 5.5) analog with improved hydrothermal stability can be facilely synthesized. In this work, ERI zeolites with different Si/Al ratios (4.6, 6.4, and 9.1) are synthesized through an ultrafast route, and in particular, a high‐silica ERI zeolite with a Si/Al ratio of 9.1 is obtained by using faujasite (FAU) as a starting material. The solid‐state ^29^Si MAS NMR spectroscopic study in combination with a computational simulation allows for figuring out the atomic configurations of the Al species in the three ERI zeolites. It is revealed that the ERI zeolite with the highest Si/Al ratio (ERI‐9.1, where the number indicates the Si/Al ratio) exhibits a biased Al occupancy at T1 site, which is possibly due to the presence of a higher fraction of the residual potassium cations in the can cages. In contrast, the Al siting in ERI‐4.6 and ERI‐6.4 proves to be relatively random.

## Graphical abstract

A high‐silica ERI zeolite (Si/Al = 9.1) is synthesized using the dealuminated FAU zeolite as the starting material. The higher fraction of the residual K^+^ cations in the *can* cages preferably direct the Al insertion into the T1 site.

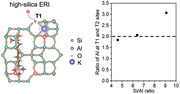

## 1 Introduction

The abatement of nitrogen oxide (NO_x_) from diesel engine exhaust remains as an urgent task in the field of environmental remediation.^[^ ^1^ ^]^ In recent years, selective catalytic reduction of NO_x_ with ammonia (NH_3_‐SCR) over metal‐exchanged zeolite catalysts has been developed as one of the most effective technologies for the mitigation of NO_x_ from mobile sources.^[^ ^2^ ^]^ In particular, small‐pore zeolites have received considerable attention due to their high hydrothermal stability and long‐lasting performance in the NH_3_‐SCR reaction.^[^ ^3^ ^]^ The implementation of the copper‐ion‐exchanged chabazite (CHA) zeolite as a commercial SCR catalyst represents a milestone success in this field.^[^ ^3e^ ^]^ Thereafter, intense efforts have been devoted to exploring materials that could potentially exhibit enhanced hydrothermal stability and improved NH_3_‐SCR activity.^[^ ^3^, ^4^ ^]^ A number of small‐pore zeolites have been investigated for this purpose, including LTA, AFX, AEI, KFI, RHO, and ERI.^[^ ^3^, ^4^, ^5^ ^]^ Typically, high‐silica zeolites are preferred for NH_3_‐SCR reaction due to their higher structural stability as compared to the low‐silica counterparts. The synthesis of high‐silica zeolites, however, usually involves the addition of complicated organic structure‐directing agents (OSDAs) or even the presence of fluoride (F^−^) media, which imposes high costs and safety concerns in obtaining high‐silica SCR catalysts.^[^ ^4^, ^6^ ^]^ It is not necessary to unlimitedly increase the Si/Al ratio of an NH_3_‐SCR catalyst, because aluminum tetrahedra are needed and important to accommodate the copper cations, which are the active sites for the catalytic reaction. Practically, there exists an optimal Si/Al ratio to balance the requirements in hydrothermal stability and the amount of redox‐active species. In addition, the hydrothermal stability as well as the catalytic performance is also governed by microenvironments of the copper species, that is, the interaction between the copper cations and the zeolite framework. Since copper cations are divalent, a pair of two neighboring framework aluminum atoms is needed to balance a Cu^2+^ cation, whereas an isolated framework aluminum site can only accommodate a copper cation in the hydrated form, [Cu(OH)]^+^. The two types of copper cations have been shown to behave differently in affecting the hydrothermal stability of the zeolite framework as well as in catalyzing the reaction.^[^ ^3^, ^7^ ^]^ Thus, the synthesis of small‐pore zeolites with tailored compositional features is of significance to designing next‐generation NH_3_‐SCR catalyst.

**Figure 1:**
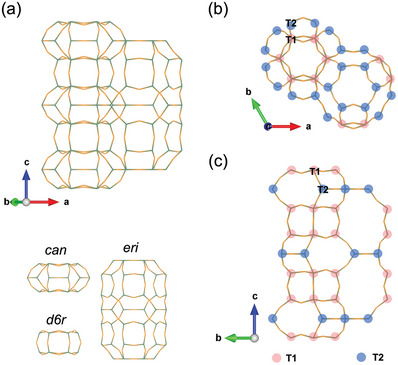
a) ERI structure and the composite building units; b) Top‐down view of ERI structure; c) Side view of ERI structure with T1 and T2 sites.

Erionite (ERI) zeolite is one of the small‐pore zeolites, which is built from erionite (*eri*) cage adjacent to a column linked by cancrinite (*can*) cage and double six ring (*d6r*) (**Figure** **1**a).^[^ ^8^ ^]^ The ERI topology belongs to the ABC‐6 family and is characterized by the AABAAC stacking sequence.^[^ ^9^ ^]^ The naturally existing ERI zeolite, however, lacks practical values owing to its low Si/Al ratio (≈3) as well as compositional complexity. An intergrowth with offretite (OFF) zeolite is generally observed in the natural form and some of the synthetic counterparts.^[^ ^10^ ^]^ ERI zeolite can accommodate different types of extra‐framework cations, including Ca^2+^, K^+^, and Na^+^, which entitles this zeolite with a high compositional versatility. The previous refinement analysis of ERI zeolites suggests that *can* cages are primarily occupied by K^+^ or Ca^2+^ in their dehydrated variety.^[^ ^11^ ^]^ The large *eri* cages are generally occupied by Na^+^, Ca^2+^, and/or Mg^2+^ cations, which are located near the six‐membered ring at the end of *eri* cage or the eight‐membered ring shared by two *eri* cages.^[^ ^11^, ^12^ ^]^ There are two topologically distinct tetrahedral (T) sites (with a multiplicity of 24 and 12, respectively) within the ERI zeolite and each unit cell contains 36 T atoms in total (T denotes Si or Al).^[^ ^10^, ^11^, ^13^ ^]^ The T1 sites are located in hexagonal prisms and the T2 sites are in the single six membered‐ring of the *eri* cage, as illustrated in Figure 1b,c.

The synthesis of a high‐silica (Si/Al>5.5) ERI‐type zeolite – UZM‐12 was first reported by the UOP researchers; thereafter, Hong and co‐workers synthesized the high‐silica ERI zeolite with a Si/Al ratio of 6.5 using diquaternary alkylammonium ions as OSDAs.^[^ ^8^, ^14^ ^]^ Although the diquaternary alkylammonium ions were used, the Si/Al ratio range of the obtained ERI zeolite was rather narrow and it was challenging to synthesize ERI zeolite with a Si/Al ratio above 7. A later success in the synthesis of SSZ‐98, an ERI‐type zeolite with a Si/Al ratio as high as 13.5, was achieved using *N,N*'‐dimethyl‐1,4‐diazobicyclo‐[2.2.2]octane as the OSDA.^[^ ^15^ ^]^ Very recently, with the aid of computational calculation, several OSDAs were screened in an attempt to synthesize high‐silica ERI zeolite. It was reported that by using cyclohexane‐1,4‐bis(trimethylammonium)dihydroxide as OSDA, ERI zeolite with a Si/Al ratio of 11 can be successfully obtained.^[^ ^16^ ^]^

The ERI zeolite holds great potential in applications such as the methanol‐to‐olefin (MTO) reaction, NH_3_‐SCR and methane partial oxidation,^[^ ^5^, ^8^, ^17^ ^]^ provided that the high‐silica ERI zeolite can be facilely synthesized. Recently, we demonstrated that an ERI zeolite (Si/Al = 5.6) obtained through an ultrafast route at high temperature (210 °C) showed excellent hydrothermal stability and catalytic performance in NH_3_‐SCR reaction when it was loaded with an optimal amount of copper.^[^ ^5c^ ^]^ As a promising material in the NH_3_‐SCR reaction, a question arises as whether the catalytic performance of ERI can be further improved by modifying the composition of the material. It is generally accepted that the Si/Al ratio not only affects the structural stability of the sample but also plays an important role in the copper locations and Brønsted acidity of the Cu‐containing catalyst. Different types and distributions of copper species are expected to form in the samples with different Si/Al ratios, which lead to varied catalytic performance. As such, for the sample with higher Si/Al ratio, [Cu(OH)]^+^ is likely to form; on the contrary, isolated copper ion Cu^2+^ predominates in the sample with lower Si/Al ratio due to the existence of Al pairs.^[^ ^7^, ^18^ ^]^ Therefore, to prepare an NH_3_‐SCR catalyst with satisfactory performance, it is of high importance to carefully tailor the chemical composition of the zeolite and tune its interaction with the copper species.

In this work, we report the synthesis of high‐silica ERI zeolite with tunable Si/Al ratios using a single OSDA. Three ERI zeolites with different Si/Al ratios (4.6, 6.4, and 9.1) can be prepared via an ultrafast route by adjusting the initial composition or the starting material in the synthesis precursor. Solid‐state ^29^Si MAS NMR spectroscopic study in combination with computational modeling were employed to reveal the atomic configurations of the Al species in the ERI zeolites of different Si/Al ratios. It turned out that the Al distribution in ERI‐9.1 is spatially‐biased as opposed to being randomly distributed in ERI‐4.6 and ERI‐6.4. The synthesis of ERI zeolites with tunable Si/Al ratios and the comprehensive analysis into their structural characteristics will deepen our understandings into the ERI zeolite and unlock its potential in industrial applications.

## 2 Results and Discussion

**Figure 2:**
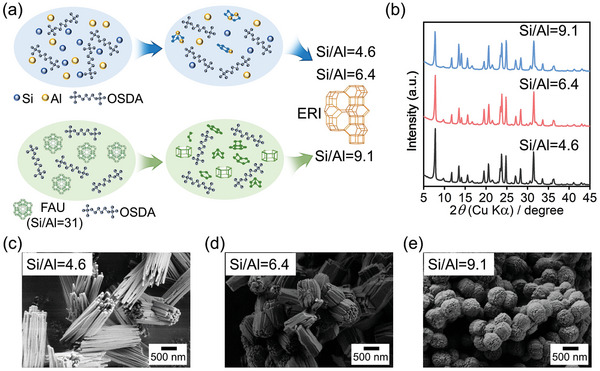
a) Illustration of the synthesis of ERI zeolites with different Si/Al ratios from amorphous Si/Al source and FAU zeolite; b) XRD patterns of ERI zeolites with different Si/Al ratios; c–e) SEM images of ERI zeolites with different Si/Al ratios.

Three ERI zeolites with different Si/Al ratios of 4.6, 6.4, and 9.1 were prepared by using either ordinary amorphous silica and alumina sources or FAU as starting material (**Figure** **2**a). The obtained samples are denoted as ERI‐*x*, where *x* indicates Si/Al ratio of the product. ERI‐4.6 and ERI‐6.4 were synthesized following the procedure reported in a previous work.^[^ ^5c^ ^]^ Both of them crystallized from the amorphous starting materials having different compositions (see Supporting Information). When we decreased the Al content in the initial reactant mixture with an attempt to further increase the Si/Al ratio, the product turned out to be of low crystallinity (Figure S1, Supporting Information). Note that in the synthesis of high‐silica zeolites, the unit cell volume of zeolite frameworks decreases with the Si/Al ratio, which generates a higher strain to the zeolite lattice and thus renders the formation of high‐silica zeolites less favorable.^[^ ^19^ ^]^ This situation explains that the synthesis of high‐silica ERI zeolite with a Si/Al ratio above 7 has been a challenge.^[^ ^8^, ^16^ ^]^ Recently, it was reported that ERI zeolites with Si/Al ratios of 13.5 and 11 can be synthesized through an interzeolite conversion from FAU zeolites, which nevertheless involved complex OSDAs.^[^ ^15^, ^16^ ^]^ Stimulated by the previous works, we explored the possibility of using a dealuminated Y zeolite (Si/Al = 31) as the starting material to synthesize the high‐silica ERI zeolite, while focusing a simple and commercially available OSDA that has been chosen for the synthesis of ERI‐4.6 and ERI‐6.4. With this strategy, an ERI zeolite with a Si/Al ratio as high as 9.1 was successfully obtained. As such, the employment of the same OSDA allowed us to readily investigate the characteristics and properties of ERI zeolites with different Si/Al ratios.

Figure 2b presents the XRD patterns of the ERI zeolites with different Si/Al ratios, showing that all the ERI samples were fully crystalline. The SEM images in Figure 2c–e depict that the three ERI zeolites exhibited distinct morphologies including needle‐like, cubic‐like, and sphere‐like shapes, although they were synthesized with the same OSDA. It is interesting to observe that the particle size of ERI products decreased with the Si/Al ratio, which is in line with the previous literature showing that the high‐silica ERI zeolite possesses smaller crystallites.^[^ ^16^ ^]^ The high crystallinity of all the three ERI samples was once again confirmed by the N_2_ adsorption–desorption isotherms (Figure S2, Supporting Information), from which a comparable micropore volume of 0.20 cm^3^ g^−1^ was obtained. ^27^Al MAS NMR spectra in Figure S3 (Supporting Information) show that only a single peak is observed at 55–57 ppm for all the three ERI samples, and no peak appears at ≈0 ppm, evidencing that the Al species were incorporated in ERI zeolite framework with tetrahedral coordination.^[^ ^8^, ^20^ ^]^ The ^27^Al MAS NMR results in accordance with the XRD patterns confirm that all the three ERI samples were highly crystalline.

**Figure 3:**
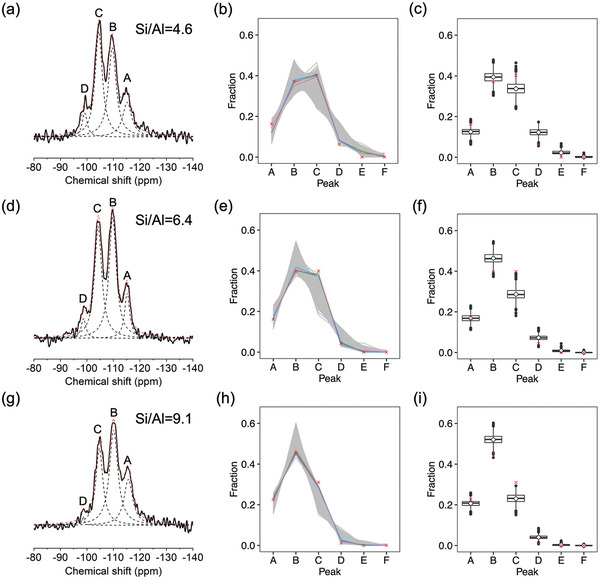
(a,d,g) shows ^29^Si MAS NMR spectra of ERI zeolites showing peaks derived from Si species coordinated with n O─Al bonds and 4‐n O─Si bonds where *n* = 0, 1, 2, 3, and 4; (b,e,h) shows box plots of the fractions of Q^4^(*n*Al) species of 4000 models with random atomic configurations; (c,f,i) shows the top five model structures (solid lines) that possess Si speciation closest to ^29^Si NMR data (cross symbols). Peak A is from T2 with Q^4^(0Al); peak B is from T1 with Q^4^(0Al) and T2 with Q^4^(1Al); peak C is from T1 with Q^4^(1Al) and T2 with Q^4^(2Al); peak D is from T1 with Q^4^(2Al) and T2 with Q^4^(3Al); peak E is from T1 with Q^4^(3Al) and T2 with Q^4^(4Al); peak F is from T1 with Q^4^(4Al).

Solid‐state ^29^Si MAS NMR measurements were performed to further investigate the local atomic coordination environments in the ERI zeolites. Particularly, deconvolution and analysis of the ^29^Si MAS NMR spectra enabled us to distinguish the differences in Al occupancy present in the three ERI samples. As demonstrated in **Figure** **3**a,d,g, the samples exhibited four major resonance peaks at ≈−114, −109, −104, and −99 ppm, which are labeled as peaks A, B, C, and D, respectively. It is well accepted that for most zeolites chemical shift dispersion in the solid‐state ^29^Si MAS NMR spectra was separated by ≈5 ppm, which is due to the distribution of aluminum species in the first coordination sphere of a SiO_4_ tetrahedron.^[^ ^21^ ^]^ Depending on the number of aluminum tetrahedral sharing oxygens with the SiO_4_, five different units corresponding to **Si**(OSi)_4_, **Si**(OAl)(OSi)_3_, **Si**(OAl)_2_(OSi)_2_, **Si**(OAl)_3_(OSi), and **Si**(OAl)_4_ species can be assigned.^[^ ^21^, ^22^ ^]^ According to the general practice, the four peaks in Figure 3 should be assigned to **Si**(OSi)_4_, **Si**(OAl)(OSi)_3_, **Si**(OAl)_2_(OSi)_2_, and **Si**(OAl)_3_(OSi) species (also called as Q^4^(*n*Al) species, where *n* can be 0 or an integer up to 4). In such a way, however, the (Si/Al)_NMR_ ratios of the three ERI samples derived from the deconvoluted spectra should be 2.9, 3.1, and 3.5, apparently lower than the values determined by inductively‐coupled plasma (ICP) measurements (4.6, 6.4, and 9.1). This inconsistency indicates that the peak assignment is not that straightforward. In fact, such a discrepancy has been observed in previous literatures.^[^ ^8^, ^23^ ^]^

One possible interpretation is that the crystallographic inequivalence in the ERI structure can also cause chemical shifts, thus complicating the peak assignment.^[^ ^21^, ^22^, ^23^, ^24^ ^]^ To test this hypothesis, we employed a well‐established empirical correlation between chemical shifts of ^29^Si MAS NMR and coordination environments of the focused Si atom in zeolites^[^ ^25^ ^]^:

(1) $$\delta \;=\;-7.2+5n-223.9\frac{\cos\bar{\alpha }}{\cos\bar{\alpha }-1}$$

*n* is the number of Al and $\bar{\alpha }\;$ is the average Si─O─T angles around the Si site. The prediction of Q^4^(0Al) in structurally optimized ERI models using this equation resulted in values of −109 ppm for T1 site and −114 ppm for T2 site. These values are strikingly consistent with peaks B and A, respectively. This also indicates that the chemical shift caused by crystallographic inequivalence is coincidentally similar to that caused by the addition or loss of an Al atom around the SiO_4_ tetrahedron. Applying the equation for various coordination environments shows that peak A is from T2 with Q^4^(0Al); peak B is from T1 with Q^4^(0Al) and T2 with Q^4^(1Al); peak C is from T1 with Q^4^(1Al) and T2 with Q^4^(2Al); peak D is from T1 with Q^4^(2Al) and T2 with Q^4^(3Al); peak E is from T1 with Q^4^(3Al) and T2 with Q^4^(4Al); peak F is from T1 with Q^4^(4Al). **Table** **1** presents the fraction and composition of Q^4^(*n*Al) for the three ERI samples with different Si/Al ratios. While this makes the interpretation of ^29^Si NMR spectra more complex, it also offers a unique opportunity to distinguish features in terms of Al occupancy if the data analysis of peak deconvolution is carefully performed.

**Table 1:** Fraction and composition of Q^4^(*n*Al) for ERI zeolites.

Sample	A^[^ ^a^ ^]^	B	C	D
		Q^4^(0Al) T1	Q^4^(1Al) T1	Q^4^(2Al) T1
	Q^4^(0Al) T2^[^ ^b^ ^]^	Q^4^(1Al) T2	Q^4^(2Al) T2	Q^4^(3Al) T2
ERI‐4.6	16.3%^[^ ^c^ ^]^	37.1%	40.3%	6.3%
ERI‐6.4	16.5%	40.4%	40.7%	4.4%
ERI‐9.1	22.6%	45.3%	31.0%	1.1%

^a)^ Deconvolution peak in ^29^Si MAS NMR spectra.

^b)^ Composition of each deconvoluted peak.

^c)^ Calculated from the peak area of each deconvoluted peak.

To figure out whether the above assignment can actually fix the discrepancy between ^29^Si NMR results and chemical analyses, we computationally generated 4000 aluminosilicate structure models for each ERI zeolite based on their chemical compositions (Si/Al ratio) measured by ICP. For each ERI zeolite, an appropriate number of Al atoms were randomly placed,^[^ ^20^, ^26^ ^]^ while abiding by the L$\ddot{o}$ wenstein's rule.^[^ ^27^ ^]^ Taking into account the chemical shifts caused by both chemical variation and crystallographic inequivalence, we were able to calculate the corresponding ^29^Si MAS NMR spectra for the computationally generated models. Figure 3b,e,h, summarize the statistical analyses of all the possible fractions for each of the three ERI zeolites. The results indicate that the fraction of Q^4^(*n*Al) species could vary significantly depending on the occupancy of the Al atoms, even though the chemical composition remains identical for the models of the same Si/Al ratio. Experimentally observed peak fraction was on the distribution of data points, indicating that the peak assignment can reconcile the NMR results and chemical analysis results without contradiction.

**Figure 4:**
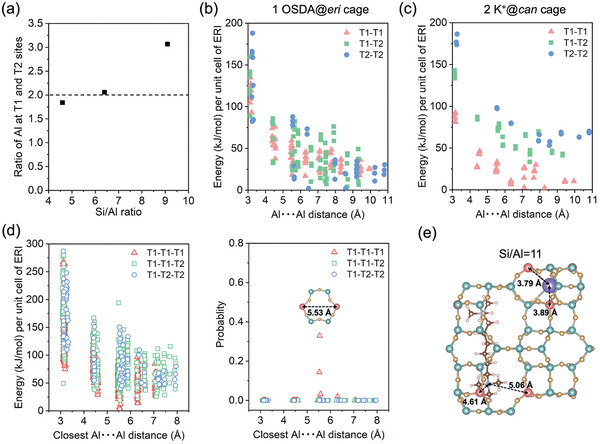
Ratio of Al at the two T sites in ERI zeolites with different Si/Al ratios; Calculated energies for Al configurations charge‐balanced by b) one OSDA molecule occluded in ERI zeolite (Si/Al = 15) and c) two K^+^ in can cages (Si/Al = 15); d) Calculated energies and probability for Al configurations with one OSDA molecule occluded in ERI zeolite and one K^+^ in can cage (Si/Al = 11); e) ERI structure having the lowest energy with Al present in T1 sites. The green and pink spheres denote Si and Al, and the purple and blue spheres denote K^+^ and N, respectively.

To understand the atomic configurations of ERI zeolites, we selected the best five models that most resemble the experimentally observed peak fractions, as shown in Figure 3c,f,i. Further, Figure S4 (Supporting Information) depicts the best crystal structures for the three ERI zeolites, from which the occupancy of Al species in the two crystallographic sites could then be derived for each ERI zeolite. As demonstrated in **Figure** **4**a, the Al distributions at T1 and T2 sites of ERI‐4.6 and ERI‐6.4 were close to 2, which match the theoretical T1/T2 ratio governed by their multiplicities (24:12).

This result indicates that the Al species in the two samples were rather randomly distributed. In contrast, the best‐matching crystallographic model of ERI‐9.1 gave a T1/T2 ratio of 3.1, which is much higher than the theoretical value, suggesting that ERI‐9.1 probably exhibited a spatially‐biased Al occupancy at the T1 site.

To figure out the origin of the biased Al configuration in ERI‐9.1, we carefully checked the chemical composition of ERI‐9.1 and compared it with those of the other two zeolites. In particular, we paid a special attention to the amounts of OSDA molecules and potassium cations in the ERI zeolites, as these organic and inorganic cations counter‐balance the Al species and may critically influence its distribution.^[^ ^20^, ^26^, ^28^ ^]^ The K^+^/Al ratios in the as‐synthesized samples were determined as 0.75, 0.62, and 0.50 for ERI‐4.6, ERI‐6.4, and ERI‐9.1, respectively (**Table** **2**). These potassium cations (K_as made_) are supposed to be located in both of the *eri* and *can* cages.^[^ ^10b^ ^]^ We carried out ammonia ion‐exchange to see the exchangeability of the potassium cations and found that some of the potassium cations could be exchanged out (K_free_), whereas a certain portion remained even after the thorough ammonia ion‐exchange. Considering the topology of the ERI structure, the exchangeable potassium cations should be located in the *eri* cage, and those unexchangeable are assumed to be locked inside the *can* cage, which were introduced during the synthesis of ERI zeolite.^[^ ^5^, ^10^, ^13^, ^29^ ^]^ To verify the energetically favorable location for K^+^ cation, we performed a Density Functional Theory (DFT) calculation and analyzed the distances from the K^+^ cations at different locations to the neighboring Al at T1 or T2 sites. The potential energies were computed over a unit cell of ERI, which contained one Al and one K^+^ cation located respectively at single eight‐membered ring (*s8r*), double six‐membered ring (*d6r*), single six‐membered ring (*s6r*), and *can* cage (Figure S5, Supporting Information). This simplified model was selected to investigate all possible locations for the Al atom. The lowest energy (defined as 0 kJ mol^−1^) was observed for the combination of K^+^ cation at *can* cage and Al occupied at T1 site (Figure S5d, Supporting Information), which gave a K^+^…Al distance of 3.80 Å. This observation suggests that the unexchanable K^+^ cations in the *can* cage (K_can_) direct Al into more energetically favorable T1 sites, resulting in a preferable occupancy of Al at the T1 sites.

**Table 2:** Chemical composition of the as‐made ERI zeolites.

Sample	Si/Al^[^ ^b^ ^]^	K_as made_ / Al^[^ ^c^ ^]^	K_can_/ Al^[^ ^d^ ^]^	K_free_/ Al^[^ ^e^ ^]^	Al/ u.c.^[^ ^f^ ^]^	K_free_/ u.c.^[^ ^g^ ^]^	K_can_ /K_free_	OSDA/ u.c.	OSDA/Al
ERI‐4.6^[^ ^a^ ^]^	4.6	0.76	0.22	0.54	6.4	3.47	0.41	1.03	0.16
ERI‐6.4	6.4	0.62	0.22	0.40	4.9	1.94	0.55	1.44	0.29
ERI‐9.1	9.1	0.50	0.24	0.26	3.6	0.93	0.92	1.77	0.49

^a)^ ERI‐*x*, *x* represents Si/Al ratio.

^b,c,d)^ Measured by ICP‐AES, where K_as made_ and K_can_ denote the K^+^ in the as‐made ERI zeolites, and the part in the *can* cages, respectively.

^e)^ K_free_ means the part of K^+^ located in the *eri* cage that can be ion‐exchanged by NH_4_^+^_._

^f,g)^ Calculated on the basis of the fact that there are 36 T atoms in per unit cell. u.c. denotes unit cell.

Furthermore, the influence of the OSDA molecules was examined. Thermogravimetry‐differential thermal analysis (TG‐DTA) was carried out to calculate the amount of OSDA molecules incorporated in the three ERI zeolites. As shown in Figure S6 (Supporting Information), the weight loss <200 °C was due to the dehydration of the sample, while that between 200 and 600 °C was ascribed to the decomposition of the OSDA molecules. Considering the size of the OSDA molecules, they should be located in the cavity of *eri* cage.^[^ ^4^, ^16^ ^]^ From the TG‐DTA results, it was calculated that ≈1.77 OSDA molecules were incorporated per unit cell, corresponding to 0.88 OSDA molecules per *eri* cage, for ERI‐9.1 (Table 2). This number is apparently higher than those in the other two samples (1.03 OSDA per unit cell for ERI‐4.6 and 1.44 for ERI‐6.4), indicating that more OSDA molecules were required to stabilize the structure with a higher Si/Al ratio. In order to study how Al was stabilized by the OSDA, we constructed a model of a unit cell of ERI, incorporating two Al atoms and one OSDA molecule within an *eri* cage. The geometry of this model was subsequently optimized using a DFT calculation. This streamlined model again facilitated the computation of energies for all feasible Al configurations that are charge‐balanced by the OSDA molecule. Figure 4b compares the calculated energies with Al distributed over the two T sites. All three combinations (T1‐T1, T1‐T2, and T2‐T2) gave a broad energy range, implying that the OSDA occluded in *eri* cages direct Al randomly. On the contrary, with two K^+^ occupied in *can* cages, Al species are distributed into more energetically favorable T1 sites (Figure 4c). These results suggest that the K^+^ cations exhibit a much stronger site‐directing ability as compared to the OSDA molecules occluded in ERI zeolite. To confirm that this observation was not a trend inherent to the chemical composition, a similar calculation was executed on a unit cell of ERI containing three Al atoms with one OSDA and one K^+^. Since the exploration of all possible locations for the K^+^ and three Al atoms is computationally demanding, we fixed the K^+^ within the *can* cage and an Al atom at the cage's T1 site, as confirmed by Figure S5 (Supporting Information). We then investigated all possible locations for the remaining two Al atoms. As shown in Figure 4d, T1 was the most stabilized crystallographic location for Al. In particular, the configuration of T1‐T1‐T1 with Al sitting at para position in the *d6r* created the most stable electronic structure (defined as 0 kJ mol^−1^) and gave the highest probability among the calculated configurations. In the ERI structure model with the most stable Al configuration (Figure 4e), the N⋯O(Al) distances observed for hexamethonium bromide and T1 were 4.61 and 5.06 Å, respectively. The values for K^+^ and T1 were calculated as 3.78 and 3.89 Å, respectively, which can likely enhance the overall stabilization. The correlation between thermodynamic stability of Al at different T sites and Al occupancy described here reveals that for ERI zeolites the Al location is primarily governed by the K^+^ cations located in *can* cage.

The synthesis of ERI zeolite with a high Si/Al ratio offers a chance to explore this small‐pore zeolite as a potential NH_3_‐SCR catalyst. To this end, the three ERI zeolites were ion‐exchanged into corresponding copper‐form ones of different Cu/Al ratios, and the resultant Cu‐ERI catalysts were labeled as Cu‐ERI‐*x*‐*y*, where *x* and *y* represent the Si/Al ratio and the Cu/Al ratio, respectively. Temperature‐programmed desorption of ammonia (NH_3_‐TPD) results over the three H‐ERI samples indicate that the desorption temperature shifted to higher temperature and the intensity of the desorption peak increased with decreasing the Si/Al ratio, which is in accordance with the previous studies (Figure S7a, Supporting Information).^[^ ^7^, ^30^ ^]^ Figure S7b (Supporting Information) further compares the NH_3_ desorption states on the Cu‐ERI samples. A new NH_3_ desorption state was observed between 300 and 400 °C, which is ascribed to NH_3_ desorption from Cu^2+^ sites in the Cu‐ERI zeolites. The comparison between NH_3_‐TPD curves for H‐ERI and Cu‐ERI zeolites suggests that after copper ion‐exchange, NH_3_ desorption from weak acid sites decreased due to the replacement with Cu^2+^ sites. In addition, the desorption temperature for NH_3_ adsorbed on Cu^2+^ sites tended to increase for the Cu‐ERI samples of lower Si/Al ratios, implying that the copper species in those samples have a stronger interaction with NH_3_. Subsequently, hydrothermal aging tests and standard NH_3_‐SCR tests were carried out over the Cu‐ERI catalysts. The hydrothermal aging of the samples was carried out at 800 °C for 5 h, and the standard NH_3_‐SCR test was performed under a space velocity of 50 000 h^−1^ (see the details in Supporting Information). The XRD patterns of the fresh and the hydrothermally aged Cu‐ERI catalysts (Figures S8–S10, Supporting Information) demonstrate that both the Si/Al ratio and the Cu/Al ratio could affect the hydrothermal stability, although the two factors contributed to opposite directions. For all three ERI zeolites, increasing the Cu/Al ratio caused a negative effect, probably because the mobility of the copper species was detrimental to the structural intactness; whereas at any given Cu/Al ratio, the Cu‐ERI‐9.1 catalyst exhibited a remarkably higher hydrothermal stability compared to the Cu‐ERI‐4.6 and the Cu‐ERI‐6.4 counterparts. The results confirm that increasing the Si/Al ratio of the ERI zeolite is beneficial to enhancing its hydrothermal stability.

**Figure 5:**
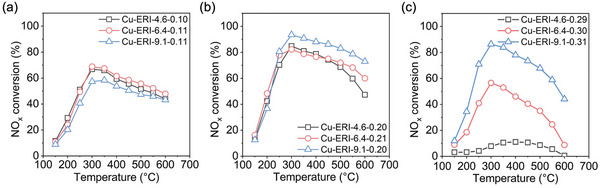
NH_3_‐SCR activity of hydrothermally aged Cu‐ERI zeolites with different Si/Al ratios but the same Cu/Al ratios. a) Cu/Al = 0.10; b) Cu/Al = 0.20; c) Cu/Al = 0.30.

In terms of the NH_3_‐SCR performance, it is of significance to find an optimal Cu/Al ratio that can balance the active sites and the hydrothermal stability. For simplicity and clarity, **Figure** **5** shows the NO_x_ conversion results of the hydrothermally aged Cu‐ERI catalysts, which were compared at identical Cu/Al ratios. At the low copper loading (Cu/Al = 0.10, Figure 5a), the aged Cu‐ERI‐9.1‐0.10 catalyst exhibited the lowest NH_3_‐SCR activity among the three catalysts compared, primarily due to the relatively low amount of copper active species. With increasing the copper loading, the benefit of a higher Si/Al ratio becomes apparent. As shown in Figure 5b,c, the aged Cu‐ERI‐9.1‐0.20 and the aged Cu‐ERI‐9.1‐0.31 catalysts remarkably outperformed the corresponding Cu‐ERI‐4.6 and Cu‐ERI‐6.4 counterparts, which is consistent with the results of hydrothermal aging (Figures S9 and S10, Supporting Information). In our previous study focusing on Cu‐ERI‐6.4, the catalyst with a Cu/Al ratio of 0.20 showed a high performance comparable to the commercial NH_3_‐SCR catalyst Cu‐SSZ‐13, which was ascribed to the optimal Cu/Al ratio at which the ERI‐6.4 could accommodate the maximum amount of isolated Cu^2+^ species. The results here show that Cu‐ERI‐9.1‐0.20 outperformed Cu‐ERI‐6.4‐0.21, indicating that the Si/Al ratio plays an overwhelming role in determining the hydrothermal stability as well as the NH_3_‐SCR activity. Furthermore, the biased Al location in the ERI‐9.1 zeolite could possibly contribute to its superior NH_3_‐SCR performance, as the preferential Al location at the T1 site may generate more Al pairs (Figure 4d); the validation of this speculation, however, could be challenging. With the above results, we can conclude that the ERI‐9.1 zeolite is a promising deNO_x_ catalyst for its superior hydrothermal stability and high NH_3_‐SCR activity.

## 3 Conclusion

In this work, we reported the synthesis of high‐silica ERI zeolites with tunable Si/Al ratios using the same OSDA, and in particular, an ERI zeolite with the Si/Al ratio as high as 9.1 was synthesized using the dealuminated FAU zeolite as the starting material. We performed the solid‐state ^29^Si MAS NMR spectroscopic study together with the computational simulation to analyze the structural characteristics, particularly, the Al siting of the high‐silica ERI zeolites. The results revealed that ERI‐9.1 exhibited a spatially‐biased Al occupancy at T1 site, probably due to the higher fraction of the residual K^+^ cations in the *can* cages, which could preferably direct the Al insertion into the T1 site. On the contrary, the Al occupancy at the two sites in ERI‐4.6 and ERI‐6.4 was found close to the theoretical ratio between the multiplicity of the two T sites, suggesting a relatively random Al distribution in the two samples. Our catalytic test results showed that due to the higher Si/Al ratio and thereof enhanced hydrothermal stability, the ERI‐9.1 zeolite outperformed the other two ERI zeolites in the NH_3_‐SCR. This study provides insight into the synthesis and the structural interpretation of high‐silica zeolite, and evidences that the high‐silica ERI zeolite holds great potential in the application as a NH_3_‐SCR catalyst.

## Conflict of Interest

The authors declare no conflict of interest.

## Supporting information

Supporting Information

## Data Availability

The data that support the findings of this study are available in the supplementary material of this article.
