# Supplementary material for: Synthesis and Structural Analysis of High‐Silica ERI Zeolite with Spatially‐Biased Al Distribution as a Promising NH3‐SCR Catalyst
Source: Adv Sci (Weinh). 2024 Feb 2;11(14):2307674. doi: 10.1002/advs.202307674 (PMC11005726; doi:10.1002/advs.202307674)
Supplement: Supplementary file 1 — Supporting Information [file ADVS-11-2307674-s001.pdf]

ADVANCED SCIENCE
Open Access

Supporting Information

for *Adv. Sci.*, DOI 10.1002/advs.202307674

Synthesis and Structural Analysis of High-Silica ERI Zeolite with Spatially-Biased Al Distribution as a Promising NH_3-SCR Catalyst

Jie Zhu, Koki Muraoka, Takeshi Ohnishi, Yutaka Yanaba, Masaru Ogura, Akira Nakayama, Toru Wakihara*, Zhendong Liu* and Tatsuya Okubo*

Synthesis and Structural Analysis of High-Silica ERI Zeolite with Spatially-Biased Al Distribution as a Promising NH_3-SCR Catalyst

Jie Zhu,[a] Koki Muraoka,*[a] Takeshi Ohnishi,[b] Yutaka Yanaba,[b] Masaru Ogura,[b] Akira Nakayama,[a] Toru Wakihara,*[a,c] Zhendong Liu*[a,c,d] and Tatsuya Okubo[a]

[a] Department of Chemical System Engineering, The University of Tokyo, 7-3-1 Hongo, Bunkyo-ku, Tokyo 113-8656, Japan

[b] Institute of Industrial Science, The University of Tokyo, 4-6-1 Komaba, Meguro-ku, Tokyo, 153-8505, Japan

[c] Institute of Engineering Innovation, The University of Tokyo, 2-11-16 Yayoi, Bunkyo-ku, Tokyo 113-8656, Japan

[d] State Key Laboratory of Chemical Engineering, Department of Chemical Engineering, Tsinghua University, Haidian District, Beijing 100084, China

Correspondence: wakihara@chemsys.t.u-tokyo.ac.jp (T. Wakihara)
liuzd@tsinghua.edu.cn (Z. Liu)
muraok_k@chemsys.t.u-tokyo.ac.jp (K. Muraoka)

Contents

1. Experimental Section

1.1 Materials

1.2 Synthesis of ERI zeolites with different Si/Al ratios

1.3 Preparation of Cu-ERI catalysts

1.4 Characterization

1.5 Computation

1.6 Catalytic reaction test

2. Supplementary Figures and Tables

Figure S1 XRD pattern of ERI zeolite synthesized by using amorphous-silica-alumina as starting material

Figure S2 N_2 adsorption isotherms of calcined ERI with different Si/Al ratios

Figure S3 ^{27}Al MAS NMR spectra of as-made ERI zeolites with different Si/Al ratios

Figure S4 Crystal structures of the ERI zeolites having Q^4(nAl) Si speciation closest to the models extracted from ^{29}Si MAS NMR

Figure S5 Calculated energies for Al configurations charged balanced by K^+ located at different positions

Figure S6 Thermogravimetry curves of ERI zeolites with different Si/Al ratios

Figure S7 NH_3-TPD curves for H-ERI and Cu-ERI-*x*-0.30 with different Si/Al ratios

Figure S8 XRD patterns of fresh and hydrothermally aged Cu-ERI zeolites with different Si/Al ratios but the same Cu/Al ratio of 0.10

Figure S9 XRD patterns of fresh and hydrothermally aged Cu-ERI zeolites with different Si/Al ratios but the same Cu/Al ratio of 0.20

Figure S10 XRD patterns of fresh and hydrothermally aged Cu-ERI zeolites with different Si/Al ratios but the same Cu/Al ratio of 0.30

1. Experimental Section

1.1 Materials

The following materials were used as received: aluminum sec-butoxide ($Al[OCH(CH_3)C_2H_5]_3$, 97%, Aldrich) as an aluminum source, colloidal silica (LUDOX® AS-40, 40 wt.% suspension) as a silica source, potassium hydroxide solution (Wako Pure Chemical Industries, Ltd., 30 wt%) as an alkali source, hexamethonium bromide (Tokyo Chemical Industry, Ltd., >98 wt%) as the organic structure-directing agent, CBV 760 (dealuminated Y zeolite with a Si/Al ratio of 31, Zeolyst International) as an alternative starting material. ERI seed crystals were synthesized according to the charge density mismatch (CDM) method.

1.2 Synthesis of ERI Zeolites with Different Si/Al Ratios

ERI zeolite synthesized with charge density mismatch (CDM) method was used as seed crystals in the fast synthesis.[1] The CDM ERI product was prepared from a reactant mixture with a composition of 6.37 TPAOH: 1.63 RBr_2: 1.43 KOH: 0.8 Al_2O_3: 16 SiO_2: 258 H_2O, where TPAOH and RBr_2 represent tetrapropylammonium and hexamethonium bromide, respectively.

The synthesis for ERI-6.4 was reported in a previous work.[1b] The initial reactant mixture had a composition of 1.63 RBr_2: 7.8 KOH: 0.8 Al_2O_3: 16 SiO_2: 258 H_2O. In a typical synthesis procedure, aluminum *sec*-butoxide ($Al[OCH(CH_3)C_2H_5]_3$, 97%, Aldrich) was dissolved in KOH solution, followed by the dropwise addition of a solution containing hexamethonium bromide dissolved in de-ionized water. Colloidal silica and de-ionized water were then added slowly to form an aluminosilicate reactant. The reactant mixture was homogenized for 2 h and aged for 20 h at 95 °C in an oven. To this aluminosilicate mixture, 10 wt% seed crystals synthesized *via* the CDM method were added, and the resulting reactant was then stirred for 10 min prior to the hydrothermal treatment. Finally, 1.7 g of the reactant mixture was fed into a tubular reactor (4.4 mm inner diameter, 6.6 mm outer diameter, 13.5 cm length) and heated at 210 °C in a preheated oil bath for 2 h. The ERI product with Si/Al=4.6 was synthesized from a reactant mixture having a composition of 1.63 RBr_2: 7.8 KOH: 1.6 Al_2O_3: 16 SiO_2: 258

H_2O. The synthesis procedure was the same as that for the synthesis of ERI with Si/Al=6.4.

For the synthesis of ERI-9.1, the initial reactant mixture had a composition of 1.63 RBr_2: 7.8 KOH: 0.27 Al_2O_3: 16 SiO_2: 258 H_2O, where CBV 760 was used as a starting material. Firstly, CBV 760 zeolite powder was dissolved in KOH solution, followed by the dropwise addition of a solution containing hexamethonium bromide dissolved in de-ionized water. De-ionized water was then added slowly to form an aluminosilicate reactant. The reactant mixture was homogenized for 2 h and aged for 20 h at 95 °C in an oven. To this aluminosilicate mixture, 10 wt% seed crystals synthesized *via* the CDM method were added, and the resulting reactant was then stirred for 10 min prior to the hydrothermal treatment. Finally, 1.7 g of the reactant mixture was fed into a tubular reactor (4.4 mm inner diameter, 6.6 mm outer diameter, 13.5 cm length) and heated at 210 °C in a preheated oil bath for 4 h.

1.3 Preparation of the H-ERI and Cu-ERI catalysts

The as-synthesized ERI zeolites with different Si/Al ratios were calcined at 550 °C for 8 h to remove the OSDAs. The calcined ERI zeolites were then ion-exchanged twice in 1 M NH_4NO_3 solution at 90 °C for 5 h to obtain the NH_4-form zeolites. The three NH_4-ERI samples were subsequently calcined in air at 550 °C for 3 h to acquire the H-ERI zeolites. To prepare the Cu-ERI catalysts, the NH_4-form ERI zeolites were ion-exchanged in a $Cu(CH_3COO)_2$ aqueous solution at 90 °C for 30 min with stirring. After centrifugation and drying in the oven, the obtained Cu-ERI catalysts were calcined at 550 °C for 3 h. The Cu-ERI catalysts with different copper loading were prepared by varying the concentration of $Cu(CH_3COO)_2$ in the aqueous solutions. Hydrothermal aging of the copper zeolites was performed at 800 °C for 5 h in a flow of air containing 10 vol.% steam.

1.4 Characterization

Powder X-ray diffraction (XRD) patterns of the catalysts were collected on a Rigaku Ultima IV X-ray diffractometer with CuKα radiation (λ = 0.15406 nm, 40 kV, 40 mA) at a scan rate of 4 °/min. The crystallinity was calculated on the basis of the areas of the

peaks ranging from 20° to 30°. Micropore volumes and BET surface areas of all the three samples were evaluated by using Quantachrome Autosorb-iQ2-MP at −196 °C. Prior to measurements, all the samples were preheated at 400 °C for 4 h under vacuum. The micropore volumes were determined by a *t*-plot method. The morphology and particle size of zeolite products were confirmed by scanning electron microscopy (JSM-7000F, JEOL, Japan) operating at an acceleration voltage of 15 keV. ^{27}Al solid state magic-angle spinning (MAS) nuclear magnetic resonance (NMR) spectra were recorded at 130.33 MHz with a $\pi/2$ pulse length of 3.2 μs, a recycle delay of 5 s, and a spinning frequency of 14 kHz. ^{29}Si dipolar decoupling (DD) MAS NMR spectra were recorded by collecting 1024 scans at 99.37 MHz with a $\pi/2$ pulse length of 5.0 μs, a recycle delay of 60 s, and a spinning frequency of 10 kHz.

Elemental analysis of the products was carried out using an inductively coupled plasma−atomic emission spectrometer (ICP-AES, iCAP-6300, Thermo) after dissolving the solid products in a hydrofluoric acid solution. Ammonia temperature programmed desorption (NH_3-TPD) was carried out using BELCAT instrument to quantify Brønsted acid sites in the H-ERI and Cu-ERI samples. The catalysts were pretreated under He environment at 500 °C for 1 h and then cooled down to 100 °C. Subsequently, ammonia adsorption was performed at 100 °C for 1 h and purged with He flow. Thereafter the temperature was raised from 100 to 750 °C with a ramp rate of 10 °C/min till NH_3 desorption is completed.

1.5 Computation

1.5.1 Atomistic models

The crystal structure of ERI zeolite was retrieved from the International Zeolite Association. The molecular structure of OSDA was optimized by the DFT calculations with B3LYP/6-31G(d,p) level of theory using Gaussian 16.[S3]

1.5.2 Interpretation of ^{29}Si MAS NMR results

In order to interpret the ^{29}Si MAS NMR results, through the identification of consistent crystal structures, the *a*- and *b*-axes of silica model, derived from the

International Zeolite Association, were doubled. This process created a supercell of ERI with 288 tetrahedral atoms. For each of the three compositions ($Si_{237}Al_{51}O_{576}$, $Si_{249}Al_{39}O_{576}$, and $Si_{259}Al_{29}O_{576}$), 4,000 models were generated by randomly substituting Si with Al in the supercell, ensuring that no Al–O–Al moiety was formed. The models that most closely matched the ^{29}Si MAS NMR spectra were selected, using the following equation as a criterion

$$\text{Difference} = \sum_i (F_i^{NMR} - F_i^{Model})$$

1.5.3 Estimating chemical shifts of ^{29}Si MAS NMR spectra

The silica model from the International Zeolite Association was structurally optimized by SLC potential using GULP 6.1.2. The resulting structure was used to estimate chemical shifts of ^{29}Si MAS NMR spectra.

1.5.4 Relative stability of 1 K

One K atom was introduced to several possible sites (*s8r*, *d6r*, *s6r* and *can*) in the unit cell of ERI. One of neighboring Si atoms was replaced with Al atom to balance the charge. These structures were then relaxed using the MPRelax setting in pymatgen version 2022.0.6, utilizing PBE functionals as implemented in VASP 6.2.1 with DFT-D3 correction. The Brillouin zone sampling was performed only at the Γ point. The energy cutoff was set to 520 eV, and the convergence criteria for the energy was set to 5×10^{-5} eV/atom.

1.5.5 Relative stability of 1 OSDA@eri

One OSDA molecule was introduced into an *eri* cage, and all potential patterns for substituting two Si atoms in the unit cell were explored. These structures were then optimized using the same method.

1.5.6 Relative stability of 2 K@can

Two potassium cations were introduced to two adjacent *can* cages aligned along the

c-axis direction. All potential patterns to substitute two Si atoms in the unit cell were generated and optimized using the same protocol.

1.5.7 Relative stability of 1 K@can and 1 OSDA@eri

Given the strong ability of K^+ to direct Al at the T1 site, a K atom was introduced into a *can* cage and an Al atom was substituted for one of the Si atoms at the T1 site within the *can* cage. Subsequently one OSDA molecule was introduced in an *eri* cage and all possible patterns to substitute two of the remaining Si atoms were enumerated. These structures were then optimized using the same method. We proceeded to compute the probability of atomic configurations, based on the assumption of the Boltzmann distribution at 423 K:

$$P_i \propto \exp\left(-\frac{E_i}{k_{\mathrm{B}}T}\right)$$

In this equation, k_B refers to the Boltzmann constant, E_i signifies the energy, and T represents the temperature.

1.6 Catalytic Reaction Test

NH_3-SCR reactions were carried out in a fixed bed reactor system equipped with a chemiluminescence (CLA) detector (VA-3000, HORIBA) as the gas analyzer. For light-off measurements, 20 mg of catalyst was mixed with 280 mg of silicon carbide, and the mixture was stuffed in a quartz reactor. A feed gas containing 300 ppm NO, 300 ppm NH_3, 5% O_2, 3% H_2O and balanced N_2 was pumped through the catalyst bed at a flow rate of 200 cm^3/min, which was corresponding to a gas hourly space velocity (GHSV) of 50,000 h^{-1}. NH_3-SCR reaction was carried out in a wide temperature range starting from 150 °C to 600 °C and the concentration of each product were detected every 50 °C after a certain period of time to ensure a steady-state condition. NO_x was calculated according to the following equation, where $[NO]_{in}$ and $[NO]_{out}$ represent inlet and outlet NO concentration, respectively.

$$NO_x \text{ conversion } \% = \frac{[NO]_{in} - [NO]_{out}}{[NO]_{in}} \times 100 \qquad (1)$$

2. Supplementary Figures

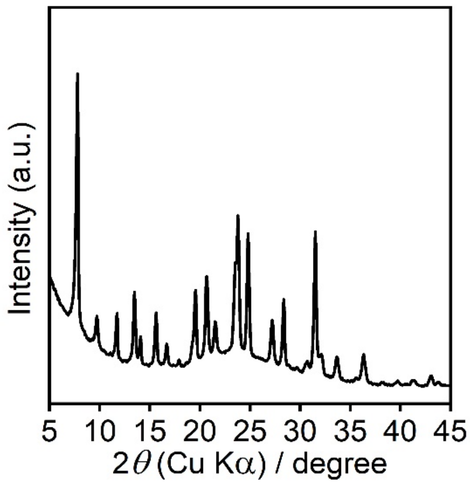

Figure S1. XRD pattern of ERI zeolite synthesized by using amorphous-silica-alumina as starting material. The Si/Al ratio of the initial reactant mixture is 31.

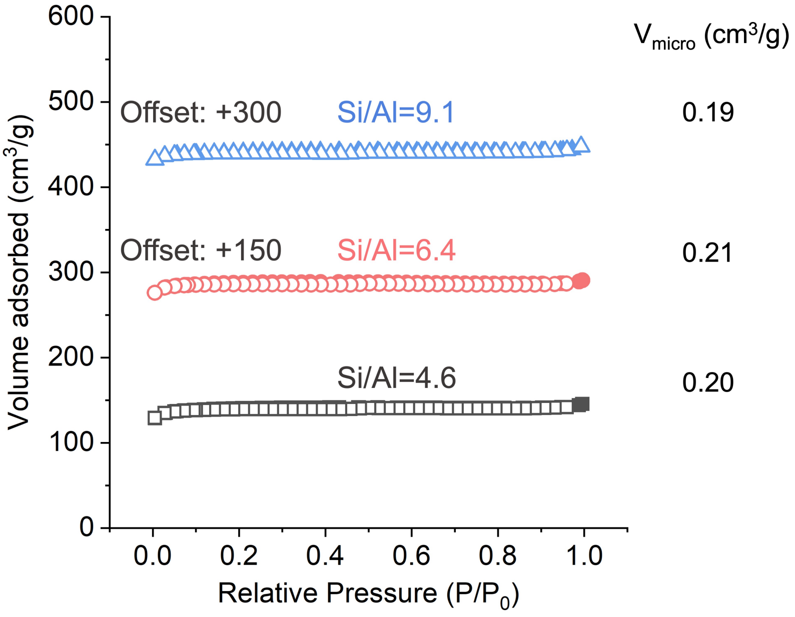

Figure S2. N_2 adsorption isotherms of calcined ERI with different Si/Al ratios.

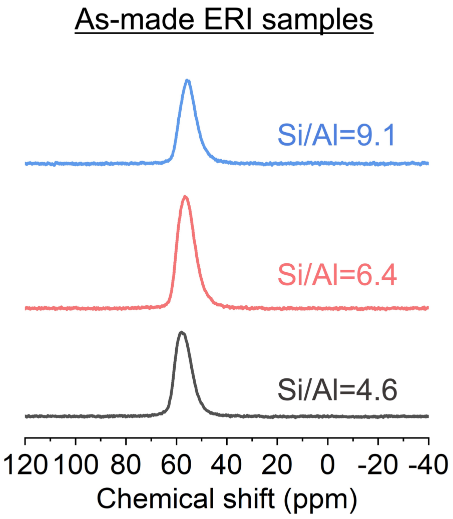

Figure S3. ^{27}Al MAS NMR spectra of as-made ERI zeolites with different Si/Al ratios.

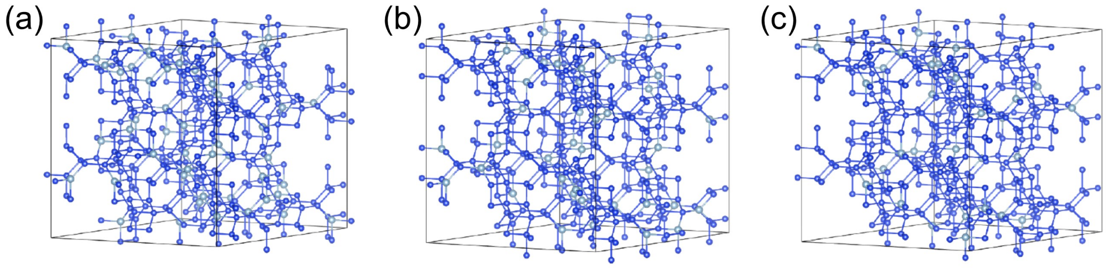

Figure S4. Crystal structures of the ERI models with (a) Si/Al=4.6; (b) Si/Al=6.4; (c) Si/Al=9.1 having Q^4(*n*Al) Si speciation closest to the models extracted from ^{29}Si MAS NMR.

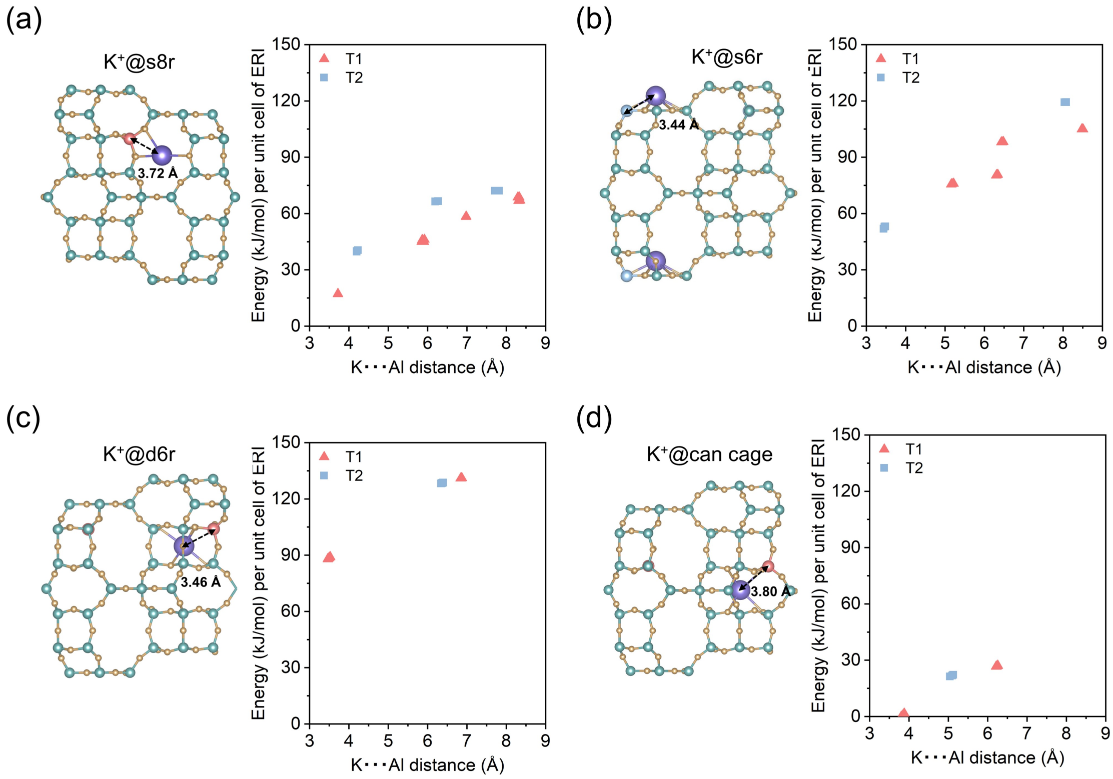

Figure S5. Calculated energies for Al configurations charged balanced by K^+ located at (a) single eight-membered ring (*s8r*), (b) single six-membered ring (*s6r*), (c) double six-membered ring (*d6r*) and (d) *can* cage in ERI zeolite with a Si/Al ratio of 35. The green and pink spheres denote Si and Al, while the purple spheres denote K.

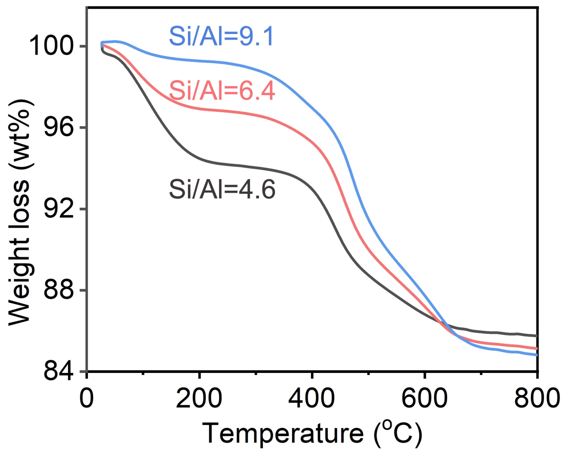

Figure S6. Thermogravimetry curves of ERI zeolites with different Si/Al ratios.

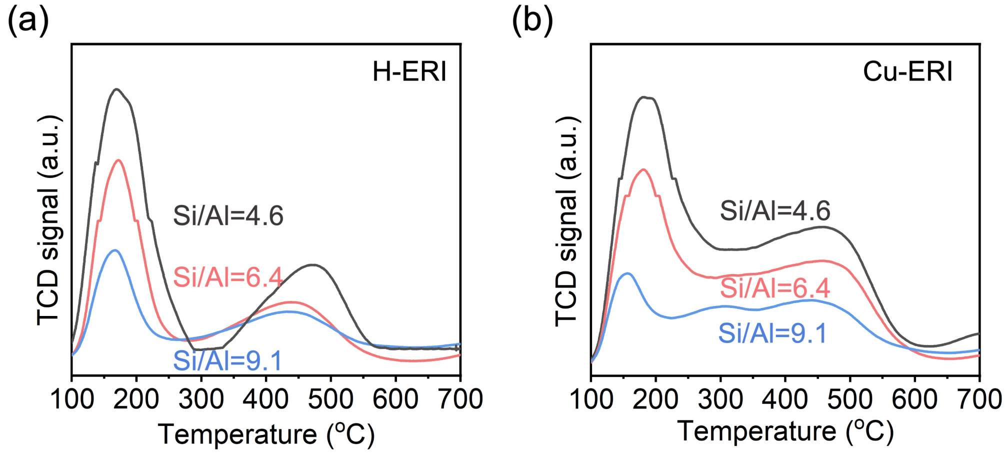

Figure S7. NH_3-TPD curves for (a) H-ERI with different Si/Al ratios; (b) Cu-ERI with different Si/Al ratios but the same Cu/Al ratio of 0.30.

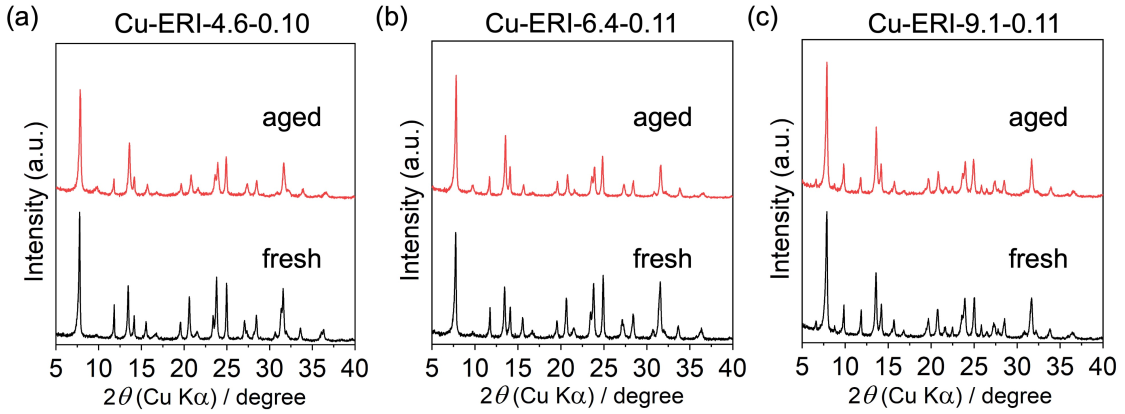

Figure S8. (a)-(c) XRD patterns of fresh and hydrothermally aged Cu-ERI zeolites with different Si/Al ratios but the same Cu/Al ratio of 0.10.

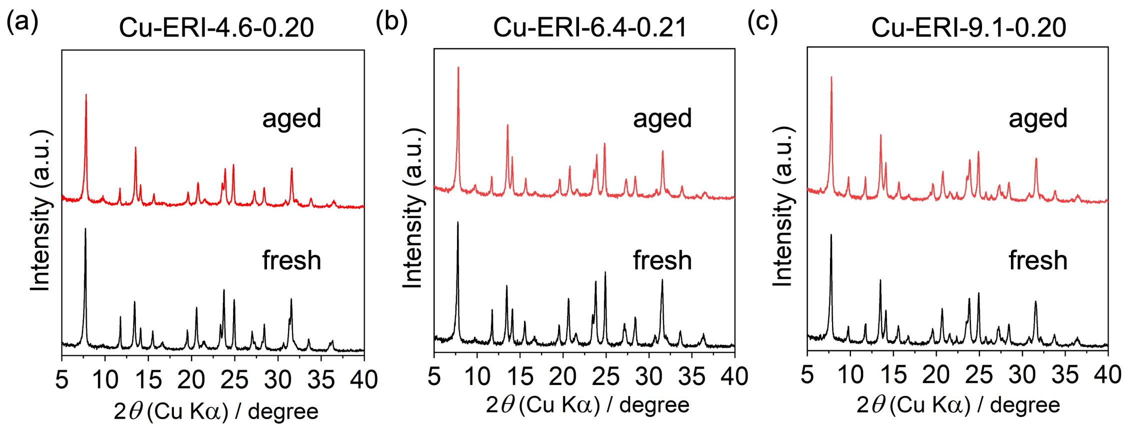

Figure S9. (a)-(c) XRD patterns of fresh and hydrothermally aged Cu-ERI zeolites with different Si/Al ratios but the same Cu/Al ratio of 0.20.

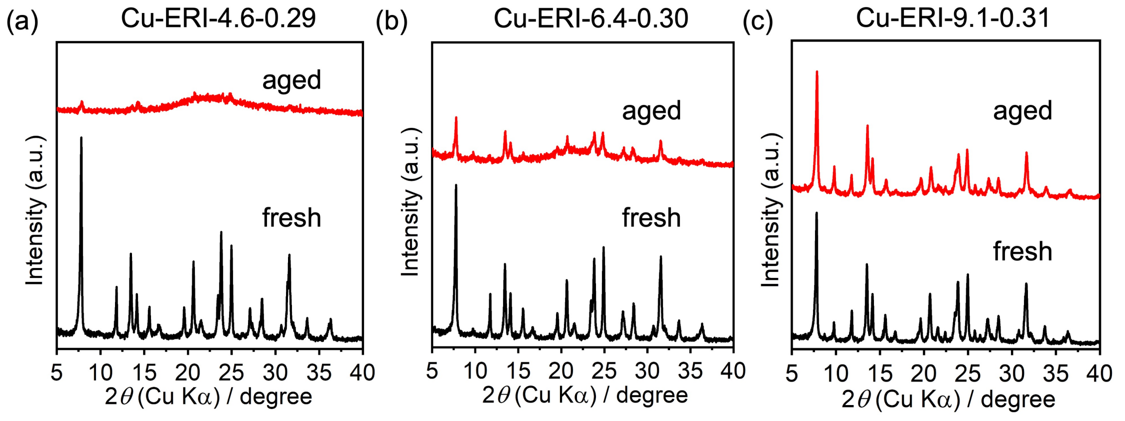

Figure S10. (a)-(c) XRD patterns of fresh and hydrothermally aged Cu-ERI zeolites with different Si/Al ratios but the same Cu/Al ratio of 0.30.

References

[S1] J. Zhu, Z. Liu, K. Iyoki, C. Anand, K. Yoshida, Y. Sasaki, S. Sukenaga, M. Ando, H. Shibata, T. Okubo, T. Wakihara, *Chem. Commun.* **2017**, *53*, 6796-6799.

[S2] J. Zhu, Z. Liu, L. Xu, T. Ohnishi, Y. Yanaba, M. Ogura, T. Wakihara, T. Okubo, *J. Catal.* **2020**, *391*, 346-356.

[S3] M. J. Frisch, G. W. Trucks, H. B. Schlegel, G. E. Scuseria, M. A. Robb, J. R. Cheeseman, G. Scalmani, V. Barone, G. A. Petersson, H. Nakatsuji, X. Li, M. Caricato, A. V. Marenich, J. Bloino, B. G. Janesko, R. Gomperts, B. Mennucci, H. P. Hratchian, J. V. Ortiz, A. F. Izmaylov, J. L. Sonnenberg, D. Williams-Young, F. Ding, F. Lipparini, F. Egidi, J. Goings, B. Peng, A. Petrone, T. Henderson, D. Ranasinghe, V. G. Zakrzewski, J. Gao, N. Rega, G. Zheng, W. Liang, M. Hada, M. Ehara, K. Toyota, R. Fukuda, J. Hasegawa, M. Ishida, T. Nakajima, Y. Honda, O. Kitao, H. Nakai, T. Vreven, K. Throssell, J. A. Montgomery, Jr., J. E. Peralta, F. Ogliaro, M. J. Bearpark, J. J. Heyd, E. N. Brothers, K. N. Kudin, V. N. Staroverov, T. A. Keith, R. Kobayashi, J. Normand, K. Raghavachari, A. P. Rendell, J. C. Burant, S. S. Iyengar, J. Tomasi, M. Cossi, J. M. Millam, M. Klene, C. Adamo, R. Cammi, J. W. Ochterski, R. L. Martin, K. Morokuma, O. Farkas, J. B. Foresman, and D. J. Fox, Gaussian, Inc., Wallingford CT, 2016.
